# Circulating Exosomes Derived-miR-146a from Systemic Lupus Erythematosus Patients Regulates Senescence of Mesenchymal Stem Cells

**DOI:** 10.1155/2019/6071308

**Published:** 2019-07-21

**Authors:** Chen Dong, Qiao Zhou, Ting Fu, Rui Zhao, Junling Yang, Xiaoli Kong, Zhongyuan Zhang, Chi Sun, Yanfeng Bao, Xinyu Ge, Zexu Zhang, Zhimin Lu, Jing Li, Wenjie Zheng, Zhifeng Gu, Juan Ji

**Affiliations:** ^1^Department of Rheumatology, Affiliated Hospital of Nantong University, Nantong, Jiangsu 226001, China; ^2^Research Center of Clinical Medicine, Affiliated Hospital of Nantong University, Nantong, Jiangsu 226001, China; ^3^Department of Stomatology, Affiliated Hospital of Nantong University, Nantong, Jiangsu 226001, China; ^4^Jiangsu Clinical Medicine Center of Tissue Engineering and Nerve Injury Repair, Affiliated Hospital of Nantong University, Nantong, Jiangsu 226001, China

## Abstract

The senescence of mesenchymal stem cells (MSCs) plays a crucial role in the development and progression of systemic lupus erythematosus (SLE). Exosomes, small spherical bilayer proteolipid vesicles, contribute to the communication between various cells and their microenvironment by transferring information via their cargo, including the proteins, lipids, and RNAs. While exosomal miRNAs participate in various biological activities, correlations of circulating exosomes with senescent signs of BM-MSCs remain unclear. In our study, we aimed at exploring the roles of circulating exosomal miRNAs in the senescence of MSCs. We found that exosomes derived from SLE serum could increase the proportions of SA-*β*-gal positive cells, disorganize cytoskeletons, and reduce growth rates. Moreover, the expression of miR-146a declined significantly in serum exosomes of SLE patients compared with healthy controls. miR-146a could be internalized into MSCs via exosomes and participate in MSCs senescence through targeting TRAF6/NF-*κ*B signaling. These results clarified the novel mechanism of MSCs senescence in SLE patients.

## 1. Introduction

Systemic lupus erythematosus (SLE) is a chronic and severe autoimmune disease that affects multiple organs with poor quality of life and substantial mortality [1]. With better knowledge of pathogenesis of SLE, glucocorticoid and immunosuppressive agents have significantly enhanced survival of SLE patients. However, some SLE patients show poor outcome and frequent relapses, which presents a challenge to rheumatologists [2]. Recently, mesenchymal stem cell transplantation (MSCT) has been regarded as a safe and effective therapeutic approach. While allogenic MSCT conferred obvious therapeutic effects on SLE by previous studies, autologous MSCT was ineffective [3–5]. Our previous studies demonstrated that SLE BM-MSCs exhibited senescent characteristics and played a vital role in SLE. Reversing the senescent phenotype of BM-MSCs could improve therapeutic effects of autologous MSCT in SLE [6, 7]. Therefore, exploring the possible senescence mechanisms of BM-MSCs is of great importance. We previously reported that lipopolysaccharide (LPS) could accelerate senescence of dental pulp stem cells (DPSCs) by NF-*κ*B-p53/p21 signaling pathway [8]. The stimulation of IFN-*γ* could promote cellular senescence in normal BM-MSCs by activating Jak-Stat signaling [9]. Similarly, serum from SLE patients obviously promoted umbilical cord (UC) derived-MSCs senescence [10]. These data suggested that inflammation in microenvironment might participate in the senescence of MSCs, and we are required to further study and investigate the exact mechanism.

Exosomes, abound in biological fluids, including serum, contribute to the communication between various cells and their microenvironment through transferring information via their cargo, including proteins, DNAs, and RNAs [11]. Increasing evidence suggests that exosomal pathway may be an efficient way to regulate cellular apoptosis, angiogenesis, and inflammatory response in target cells [12]. Previous study showed that exosomes derived from cancer cells in multiple myeloma patients could regulate the proliferation of MSCs via modulating miR-146a and miR-21 [13]. MicroRNA (miRNA), a small noncoding RNA molecule, induces translation repression of the target mRNA and influences cellular metabolism, proliferation, and apoptosis [14]. In the study, we devote ourselves to investigating the effects and potential mechanisms of serum exosomes derived-miRNAs on BM-MSC senescence in SLE.

## 2. Materials and Methods

### 2.1. SLE Patients

The diagnosis of all SLE patients was dependent on the criteria determined by the 2009 American College of Rheumatology (ACR) (Table 1). Systemic lupus erythematosus diseases activity index (SLEDAI) was used to evaluate SLE disease activity according to clinical manifestation and laboratory examination. 10 healthy female donors (27.2±7.8 years) and 10 female SLE patients (26.9±7.3 years) were enrolled in this study. Serum samples and BM-MSCs were extracted from the participants. All participators gave written consent to the study, which was approved by the Ethics Committee of Affiliated Hospital of Nantong University (Nantong, China).

### 2.2. Isolation and Cell Culture of BM-MSCs

BM-MSCs, derived from heparinized BM of healthy donors and SLE patients, were isolated by using Ficoll PREMIUM (1.073g/ml). The BM-MSC layer was collected, washed by phosphate-buffered saline (PBS) three times, then plated in T25/75 cell culture flask, and cultured at 37°C, in 5% CO2 incubator. After 5 days, the medium was replaced every 3 days until the cells reach 80% confluence. The culture medium was Low-glucose Dulbecco Modified Eagle Medium (L-DMEM) with 10% heat inactivated fetal bovine serum (FBS) (Gibco, Carlsbad, USA), 10% normal serum or SLE serum. When BM-MSCs became nearly 80% confluent, the adherent cells were digested with 0.25 % trypsin–EDTA (Gibco, USA) and replated at a density of 1×10^6^ cells per T25 flask.

### 2.3. Extraction of Exosomes from Serum

Serum samples were centrifuged to get rid of cellular debris. The cell-free serum supernatant (200ul) was transferred to a fresh tube and each sample was blended with 1/5 volume (40ul) of Total Exosome Isolation from serum reagent (Invitrogen, USA). The samples were centrifuged at 10,000g at room temperature for 10 min after being incubated at 4°C for 30 min. The supernatant was aspirated and discarded, and the exosome bullet was resuspended in PBS buffer (200ul). It was stored at 4°C for short term (less than 7 days) or 20°C for longer term. The concentration of exosomes was measured by BCA protein assay kit (Beyotime, Shanghai, China).

### 2.4. Characterization of Exosomes

After being fixed with 2.5% glutaraldehyde, isolated exosome bullets were centrifuged at 100 000g to remove the glutaraldehyde. The bullets were negatively stained by 3% aqueous phosphotungstic acid and fixed on copper mesh Formvar grids. The morphology of exosomes samples was observed by the JEOL Transmission Electron Microscope (JEM-1230; JEOL, Tokyo, Japan). The size of exosomes was detected by nanoparticle tracking analysis (NTA), using ZetaView PMX 110 (Particle Metrix, Meerbusch, Germany) and corresponding software ZetaView 8.04.02.

### 2.5. Exosomes Uptake Assay

Cellular uptake assay of serum exosomes was measured using the PKH-67 labeling kit (Sigma, USA). Exosomes bullets were resuspended in 300ul Diluent C, and then exosomes suspension was incubated with 4 *μ*l PKH-67 dye diluted in 300ul Diluent C at room temperature. After 5 minutes, the suspension was mixed with in 3ml FBS and centrifuged at 100,000g to pellet the PKH-67-labeled exosomes. After incubation with exosomes solution for 3h, BM-MSCs were fixed in 4% paraformaldehyde for 30 minutes at room temperature. The cell nuclei were dyed with DAPI (Sangon Biotech, Shanghai). BM-MSCs were detected by a fluorescence microscope (Leica, Germany).

### 2.6. Western Blot Assay

Equal parts of proteins were separated by SDS polyacrylamide gel electrophoresis and electrophoretically transferred to polyvinylidene difluoride (PVDF) membranes. Membranes were blocked with 5% bovine serum albumin and then incubated with primary antibodies against p-p65, p65, p-I*κ*B*α* (Cell Signaling Technology, USA), p53, CD63, p27, CD9 (Sangon Biotech, Shanghai) at 4°C overnight. After being washed 3 times, samples were incubated with the corresponding horseradish peroxidase-conjugated secondary antibody (Cell Signaling Technology, USA) for 2 h at room temperature. The blots were visualized by using an enhanced chemiluminescence kit (Millipore Corporation, USA).

### 2.7. SA-*β*-Gal Assay

The senescence-associated *β*-galactosidase (SA-*β*-gal) activity of BM-MSCs was measured by a kit from the Chemical Company (Beyotime, China). Following the manufacturer's instructions, MSCs were cultured into the six-well culture plates at a density of 5 × 10^4^ cells per well. Then cells were washed with PBS and fixed with fixation solution. After incubation with SA-*β*-gal staining solution at 37°C without CO_2_ overnight, BM-MSCs were washed with PBS and analyzed by using the microscope.

### 2.8. Immunofluorescent Staining

After being fixed with 4% paraformaldehyde for 40-60 minutes, BM-MSCs were washed with PBS containing 0.1% Triton X-100(PBST) and blocked for 30 minutes in PBST supplemented with 10% FBS. BM-MSCs were incubated with the primary antibodies (1:100) in the same solution at 4°C overnight. The primary antibodies were p65 and I*κ*B*α* (Cell Signaling Technology, USA). Then, BM-MSCs were washed and incubated in secondary antibodies at room temperature for 2 h. The cell nuclei were stained with DAPI (Sangon Biotech, Shanghai). The cells were visualized using a Leica fluorescence microscope (Germany).

### 2.9. Immunofluorescence Assay of the Skeleton of MSCs

BM-MSCs were washed with PBS and fixed with 4% paraformaldehyde for 40-60 minutes. After permeabilization and block, BM-MSCs were incubated with fluorescein isothiocyanate-conjugated phalloidin (Thermo Fisher, Waltham, USA). The cell nuclei were stained with DAPI (Sangon Biotech, Shanghai). The stained cells were visualized with a Zeiss Confocal Laser Scanning Microscope (Oberkochen, Germany).

### 2.10. Quantitative Reverse-Transcription PCR

miR-146a is a type of miRNA associated with immunity, participating in cell differentiation, cell proliferation, cell immune response, and release of inflammatory mediators [15]. Exosomal miRNA was extracted using the Total Exosome RNA Kit (Ambion) and MirVana RNA isolation kit (Ambion) following the manufacturer's instructions. U6 was used as the internal reference for qualification of the cellular miRNA. The PCR primers were purchased from Biomics Biotechnologies (Nantong, China).

### 2.11. Transient Transfection with miR-146a Mimics/Inhibitors

miR-146a-5p mimics or inhibitors and their corresponding negative controls were bought from Biomics Biotechnologies (Nantong, China) and transfected at a final concentration of 50 nM for mimics and 100 nM for inhibitor in BM-MSCs in accordance with the manufacturer's instructions.

### 2.12. Statistical Analysis

All data are expressed as the mean ± standard deviation (SD). All statistical analysis was performed by the SPSS 21.0 software, and significant differences between these data were determined by using Student's t-test.* P < *0.05 was considered statistically significant.

## 3. Results

### 3.1. SLE Serum Stimulation Promoted Senescence of MSCs

**Figure 1:**
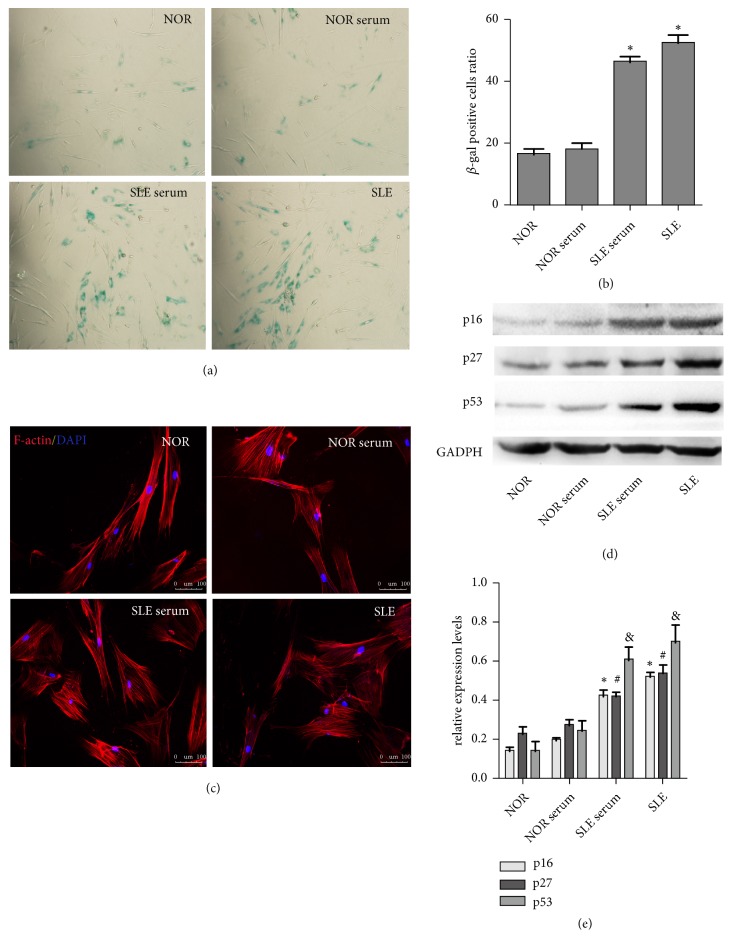
*SLE serum stimulation promoted senescence of MSCs*. (a, b) Cells were cultured in serum from normal persons and SLE patients. BM-MSCs were fixed and stained with SA-*β*-gal. The number of SA-*β*-gal-positive cells was increased in the serum-treated normal BM-MSCs in comparison with the normal group. (c) Immunofluorescence showed that the normal distribution of F-actin in the BM-MSCs from normal persons was disordered after being stimulated with SLE serum. (d, e) Western blotting analysis was performed to detect the protein expressions of p16, p27, and p53 (^∗^*P* < 0.05 compared with the normal group,^ #^*P* < 0.05 compared with the normal group, ^&^*P* < 0.05 compared with the normal group) (NOR: normal MSCs group, NOR serum: normal MSCs treated with normal human serum, SLE serum: normal MSCs treated with serum from SLE patients, SLE: SLE MSCs group) ( SLE MSCs: positive control).

Previously, our studies demonstrated that BM-MSCs in SLE patients were senescent [9]. To further explore the senescent biological behaviours of BM-MSCs stimulated by SLE serum, we detected SA-*β*-gal staining, the F-actin distribution, and the expression levels of cell cycle-related proteins (p16, p27, and p53) in BM-MSCs. We found that the ratio of SA-*β*-gal positive BM-MSCs was upregulated significantly in the group treated with SLE serum, compared with ones treated with normal serum (Figures 1(a) and 1(b)). The F-actin distribution was disordered in MSCs treated with SLE serum (Figure 1(c)). Additionally, the expressions of p16, p27, and p53 proteins were increased in SLE serum-handled BM-MSCs by western blotting (Figures 1(d) and 1(e)). It suggested that SLE serum could enhance the senescence signs of MSCs* in vitro*, which might play a significantly vital role in MSCs senescence.

### 3.2. Serum Exosomes from SLE Patients Enhanced the Senescence of MSCs by Activating NF-*κ*B Signaling Pathway

**Figure 2:**
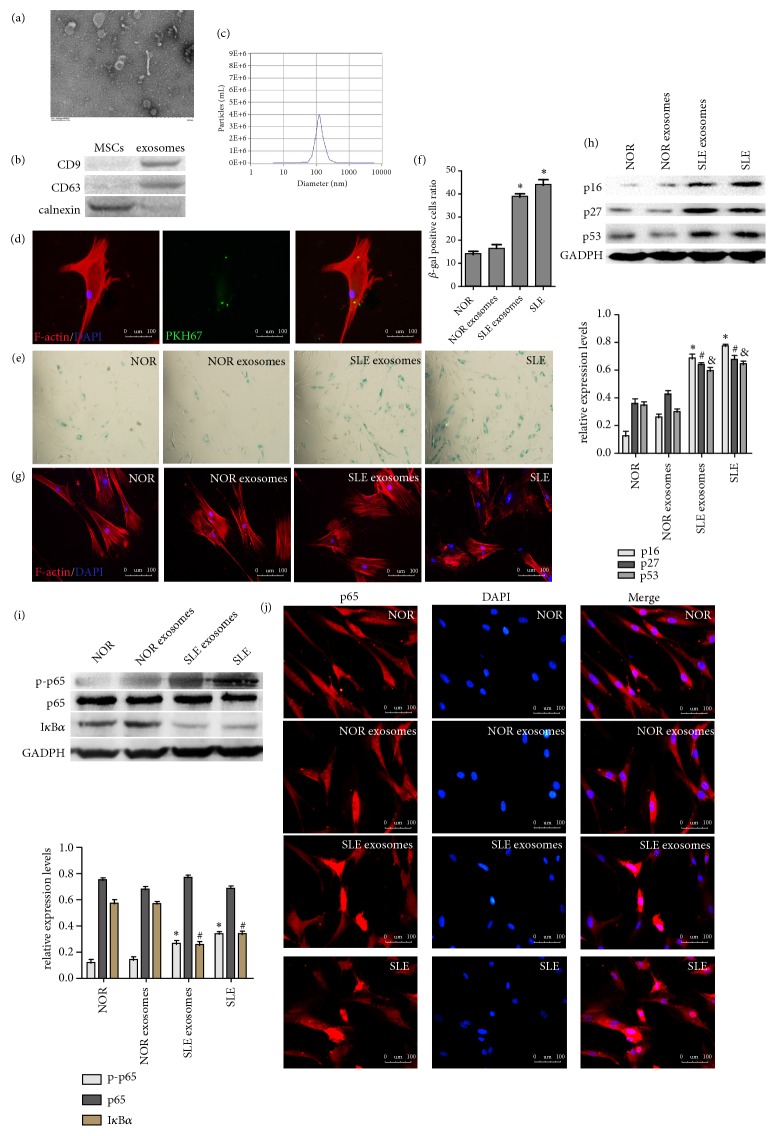
*Serum exosomes from SLE patients enhanced the senescence of MSCs by activating NF-κB signaling pathway*. (a) Serum exosomes were observed under a transmission electron microscope. (b) Western blotting analysis of CD9, CD63, and calnexin expression in lysates from purified serum exosomes. (c) Nanoparticle tracking analysis was used to detect the size distribution of exosomes. (d) BM-MSCs were incubated in serum exosomes that were labeled with PKH67 (green). (e, f) The number of SA-*β*-gal-positive cells was increased in the SLE serum exosomes-treated BM-MSCs in comparison with the normal group. (g) Immunofluorescence showed that distribution of F-actin in the BM-MSCs was disordered after being stimulated with SLE exosomes. (h) The protein expressions of p16, p27, and p53 were increased in MSCs treated with SLE serum exosomes by western blotting analysis. (i) Western blot analysis was performed to examine the protein expressions of p-p65, p65, I*κ*B*α*. (j) Immunofluorescence staining of p65 in BM-MSCs treated exosomes (^∗^*P *< 0.05 compared with the normal group,^ #^*P* < 0.05 compared with the normal group, ^&^*P* < 0.05 compared with the normal group) (NOR: normal MSCs group, NOR exosomes: normal MSCs treated with normal human exosomes, SLE exosomes: normal MSCs treated with exosomes from SLE patients, SLE: SLE MSCs group).

To investigate whether exosomes mediated MSCs senescence, serum-derived exosomes were purified by ExoQuick method, it was observed that average size was 100nm in diameter, and membrane vesicles were observed by transmission electron microscopy (Figure 2(a)). CD63 and CD9, representative markers of exosomes, were detected by western blotting; calnexin (a marker of endoplasmic reticulum) was a negative control for exosomes (Figure 2(b)). Nanoparticle tracking analysis was used to detect the size distribution of exosomes (Figure 2(c)). The results demonstrated that serum-derived exosomes were purified successfully. Then, we cultured normal BM-MSCs with SLE serum exosomes for 24h. As shown in Figure 2(d), the fluorescent results showed that PKH-67-labeled exosomes could be localized in the cytoplasm of BM-MSCs. In accordance with the role of SLE serum in MSCs senescence, the ratio of SA-*β*-gal positive cells treated with SLE serum exosomes was higher than that of cells treated with normal serum (Figures 2(e) and 2(f)). Following the treatment of SLE serum exosomes, the distribution of F-actin in normal BM-MSCs presented derangement distribution (Figure 2(g)). In addition, the expressions of p16, p27, and p53 were determined in MSCs treated with SLE serum exosomes (Figure 2(h)). To explore the molecular mechanism of serum exosomes in promoting MSCs senescence, we found that SLE serum exosomes induced the degradation of I*κ*B*α* and phosphorylation of p65 (Figure 2(i)). To further examine the nuclear accumulation of p65 in BM-MSCs, immunofluorescence staining showed that p65 could translocate to the nucleus following exposure to SLE exosomes (Figure 2(j)). These results indicated that SLE serum exosomes promoted senescence of MSCs through NF-*κ*B signaling pathway* in vitro*.

### 3.3. Serum Exosomal miR-146a Regulated the Senescence of MSCs

**Figure 3:**
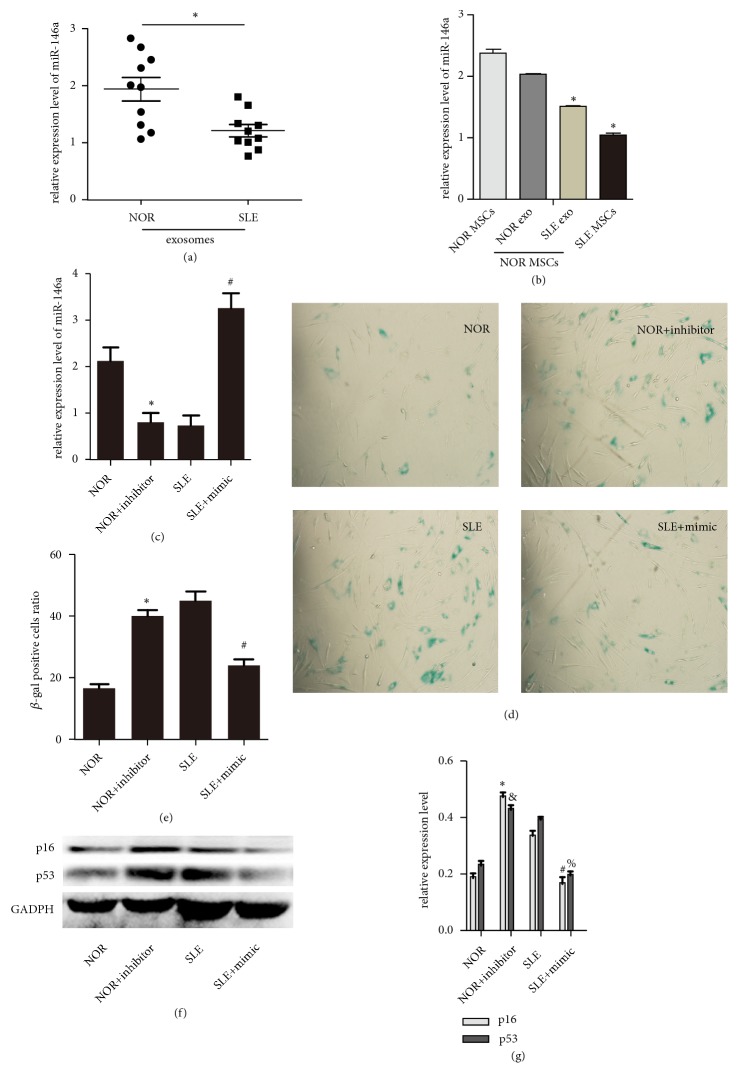
*Serum exosomal miR-146a regulated the senescence of MSCs*. (a) QRT-PCR showed the miR-146a expression in serum exosomes of normal persons and SLE patients. (b) QRT-PCR showed the miR-146a expression in MSCs treated with normal exosomes and SLE exosomes. (c) NOR MSCs were transfected with miR-146a inhibitors, and SLE MSCs were transfected with miR-146a mimics. The results showed the transfection efficiency was successful. (d, e) MSCs were fixed and stained for *β*-gal. The number of SA-*β*-gal-positive cells was obviously reversed among miR-146a mimics-treated SLE MSCs in comparison with untreated group. (f, g) The protein expressions of p16 and p53 were detected in NOR MSCs transfected with miR-146a inhibitors and SLE MSCs transfected with miR-146a mimics by western blotting analysis (^∗^*P *< 0.05 compared with the normal group,^ #^*P* < 0.05 compared with the SLE group, ^&^*P* < 0.05 compared with the normal group) (NOR: normal MSCs group, NOR exo: normal MSCs treated with normal human exosomes, SLE exo: normal MSCs treated with exosomes from SLE patients, SLE: SLE MSCs group).

Given the previous study that miR-146a levels were obviously downregulated in the PBMCs of SLE patients [16], we assessed the levels of miR-146a in serum exosomes from 10 SLE persons. Our results discovered that compared with healthy controls, the levels of miR-146a declined significantly in SLE exosomes (Figure 3(a)). The levels of miR-146a were decreased in MSCs treated by SLE exosomes (Figure 3(b)). To further discover the correlation between the expression of miR-146a and cellular senescence in SLE patients, NOR MSCs or SLE MSCs were transfected with miR-146a inhibitors or mimics, respectively. The transfection efficiency was viewed in Figure 3(c). Interestingly, we found that administration of miR-146a mimics reversed SLE MSCs senescence, as demonstrated by decreased frequencies of SA-*β*-gal positive cells (Figures 3(d) and 3(e)). Furthermore, the expressions of p16 and p53 were determined in NOR MSCs or SLE MSCs that were transfected with miR-146a inhibitors or mimics (Figures 3(f) and 3(g)). These western blotting results were consistent with previous SA-*β*-gal assay results. Taken together, these data demonstrated that miR-146a-containing exosomes might regulate the senescence of MSCs.

### 3.4. miR-146a Directly Targeted TRAF6 and Inhibited TRAF6-NF-*κ*B Signaling Expression

**Figure 4:**
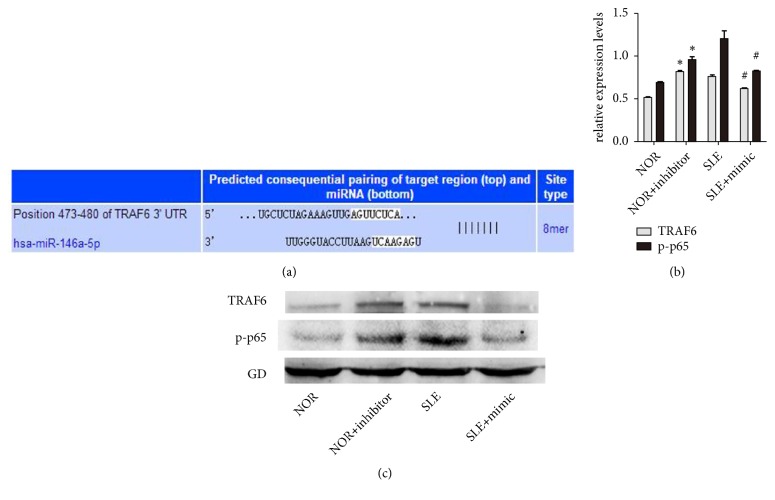
miR-146a directly targeted TRAF6 and inhibited TRAF6-NF-*κ*B signaling expression. (a) TargetScan was used to identify putative seed-matching sites between miR-146a and 3'-UTR of TRAF6. (b, c) NOR MSCs were transfected with miR-146a inhibitors, and SLE MSCs were transfected with miR-146a mimics. After being transfected, each cell was detected for TRAF6 and p-p65 expression levels (^∗^*P* < 0.05 compared with the normal group, ^*#*^*P* < 0.05 compared with the SLE group).

Previous study showed that miR-146a could negatively regulate immune inflammation by suppressing NF-*κ*B signaling pathway activation [17, 18]. According to the bioinformatic algorithms (TargetScan), TRAF6 was selected for further analysis because of its correlation with the senescence regulation of exosomes (Figure 4(a)). The transfection data showed that overexpression of miR-146a significantly decreased the expression level of TRAF6 in BM-MSCs (Figures 4(b) and 4(c)).

## 4. Discussion

**Figure 5:**
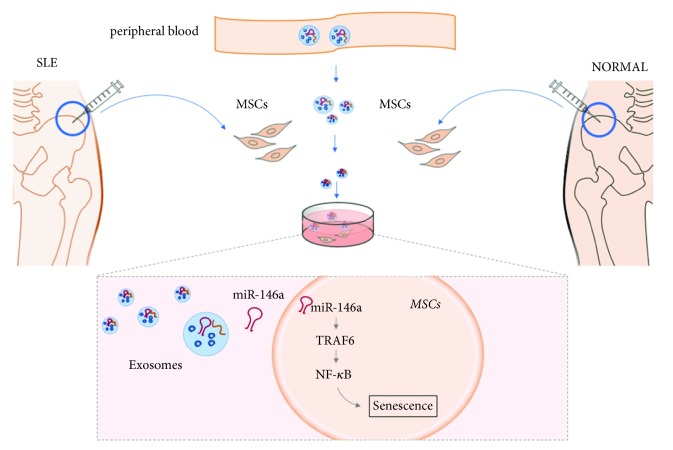
*The schematic mechanism of exosomal miR-146a from SLE patients regulating senescence of MSCs through targeting TRAF6/NF-κB signaling.* BM-MSCs were isolated from bone marrow of healthy donors and SLE patients. Serum-derived exosomes were purified by ExoQuick method and cocultured with BM-MSCs for 24h; then we analyzed the senescent status of BM-MSCs, including SA-*β*-gal staining, the F-actin distribution, and the expression levels of cell cycle-related proteins. miR-146a levels in SLE exosomes significantly declined in comparison with NOR group. miR-146a might negatively regulate MSCs senescence by suppressing TRAF6/NF-*κ*B signaling pathway activation.

In this study, we further confirmed that SLE serum stimulation could promote senescence of MSCs. In addition, serum-derived exosomes, as a tool for cell communication, could stimulate the senescence of MSCs by activating NF-*κ*B signaling. Exosomal miR-146a might negatively regulate MSCs senescence by targeting TRAF6/ NF-*κ*B signaling (Figure 5).

Previous studies have revealed that SLE is a stem cell-related disease. Senescent MSCs play an important role in SLE. Our groups further discovered that SLE MSCs exhibited senescent characteristics, the increased SA-*β*-gal staining, disordered cytoskeletons, and low-growth rates [19]. Various signaling pathways are involved in MSCs senescence, including PI3K/Akt pathways, Wnt/*β*-catenin signaling, and NF-*κ*B pathway. Of them, NF-*κ*B signaling pathway achieves very prominent positions in senescent MSCs [20]. The NF-*κ*B pathway transcriptionally controls a large amount of target genes that contribute to cellular inflammation and senescence [21]. Our group found that NF-*κ*B/p53/p21 signaling pathway participated in the senescence process of DPSC [8]. Similarly, upregulated NF-*κ*B activity in aged MSCs was also observed by a recent study [22]. In the classical pathway of NF-*κ*B signaling, p65 is localized in the cytoplasm predominantly as a complex with the inhibitory I*κ*B protein in inactivated cells. Once stimulated, p65 is shuttled from the cytoplasm to the nucleus with I*κ*B releasing from the inactive complex followed by ubiquitin-mediated proteasomal degradation of I*κ*B [23]. As we expected, western blotting showed that the expression level of p-p65 was elevated in SLE BM-MSCs. p65 translocating to nucleus was increased in SLE MSCs. Therefore, NF-*κ*B signaling was crucial for senescence of MSCs in SLE patients.

Exosomes can carry a lot of bioactive molecules, such as protein, lipid and mRNA, miRNAs, long noncoding RNAs (lncRNAs), and genomic DNA, then transfer their cargos to neighboring cells, and induce the functional modifications in recipient cells [24, 25]. Our group discovered that exosomes from SLE patients could promote MSCs senescence, displaying increased SA-*β*-gal staining and disordered cytoskeletons. Recent studies have reported that lung cancer cell-derived exosomes can accelerate MSCs to release proinflammatory cytokines, such as IL-8 and IL-6, by activating NF-*κ*B signaling of MSCs [26]. Previous study has depicted an important role for NF-*κ*B activity in facilitating senescence of human fibroblasts via the p53/p21 and p16/pRB pathway [27]. In RA patients, serum exosomal miR-548a-3p could restrain the proliferation of pTHP-1 cells by improving the TLR4/NF-*κ*B signaling pathway [28]. To ascertain whether NF-*κ*B signaling pathway was involved in the process of inducing MSCs senescence by serum exosomes, the level of p-p65 was increased in BM-MSCs treated with SLE exosomes. Immunofluorescence staining showed that SLE exosomes treatment was sufficient to increase p65 translocating to nucleus. Hence, we could conclude that serum exosomes from SLE patients enhanced the senescence of MSCs by activating NF-*κ*B signaling pathway.

The previous study provided data that endothelial cells could release exosomes containing miR-214, which could inhibit senescence and promote angiogenesis in target cell [29]. miR-146a is an immune related miRNA, participating in cell differentiation and proliferation, cell immune response, and release of inflammatory mediators [15, 18]. In the majority of many researches, miR-146a acted as a negative regulator of inflammatory signaling pathways. There was evidence showing that chronic, systemic, low-grade inflammation, including IL-1 and TNF-*α*, contributed to the development of aging [30]. Previous research showed that miR-146a was involved in the senescence of human fibroblasts in IL-1 dependent manner [31]. The level of miR-146a was obviously downregulated in the PBMCs of SLE patients, and it could restrain the expression and secretion of IL-1*β*, IL-6, IL-8, and TNF-*α* [32]. It negatively regulated NF-*κ*B signaling pathway and inflammatory reaction activation through targeting TRAF6 [33]. In this study, we observed that serum exosomal miR-146a was decreased in 10 SLE patients in a miRNA level. To further evaluate the role of miR-146a in regulating MSCs senescence, we performed SA-*β*-gal assay. miR-146a mimics could reverse the SLE MSCs senescence, suggesting that serum exosomal miR-146a regulated MSCs senescence. Furthermore, TRAF6/NF-*κ*B signaling pathway was indicated as the main functional targets of miR-146a in mediating BM-MSCs senescence phenotype.

In summary, this study was the first to report that circulating exosomes derived-miR-146a from SLE patients could promote senescence of MSCs via TRAF6/NF-*κ*B signaling pathway. Targeted-MSCs senescence might improve the transplantation efficacy of BM-MSCs in SLE patients. However, this study has some limitations; we cannot conclude that the effect of serum inducing MSCs senescence is definitively due to exosome uptake, because the remaining portion of the SLE serum might also promote MSCs senescence. The widespread method of exosomes extraction from serum is not perfect, because the remaining supernatant after isolation is the mixture of serum and exosome isolation reagent or diluted serum. In addition, the transfection of miR-146a directly into isolated exosomes needs to be further performed, and the negative effects of exosomal miR-146a mediated MSCs senescence need to be further investigated in the animal model in future study.

## 5. Conclusion

Our study demonstrated that circulating exosomes from SLE patients could induce senescence in MSC in vitro and this effect could be partially explained by the loss of miR-146a content.

## Figures and Tables

**Table 1:** Details of 10 SLE patients.

Patient	Age	Disease duration	Current treated	SLEDAI
1	21	1 year	Pred 15mg/day	10
			HCQ 0.2g/day	
2	18	9 months	Pred 15mg/day	14
			HCQ 0.2g/day	
			LEF 0.2g/day	
3	24	2 years	Pred 15mg/day	9
			HCQ 0.2g/day	
4	28	1 year	Pred 15mg/day	12
			HCQ 0.2g/day	
5	24	2 years	Pred 10mg/day	14
			HCQ 0.2g/day	
			CTX 0.4g/2 weeks	
6	32	3 years	Pred 15mg/day	12
			HCQ 0.2g/day	
7	43	6 years	Pred 10mg/day	9
			HCQ 0.2g/day	
8	21	4 days	none	13
9	26	1 month	Pred 12.5mg/day	14
			HCQ 0.2g/day	
10	32	1 year	Pred 20mg/day	21
			HCQ 0.2g/day	
			CTX 0.4g/2 weeks	

Pred: prednisolone, HCQ: hydroxychloroquine.

LEF: leflunomide, CTX: cyclophosphamide.

## Data Availability

The data used to support findings of the research are available from the corresponding author upon request.
